# PROTOCOL: A mixed methods systematic review on the effects of arts interventions for at‐risk and offending children and young people on behavioural, psychosocial, cognitive and offending outcomes

**DOI:** 10.1002/cl2.1298

**Published:** 2023-01-10

**Authors:** Louise Mansfield, Norma Daykin, Neil E. O'Connell, Daniel Bailey, Louise Forde, Robyn Smith, Jake Gifford

**Affiliations:** ^1^ Department of Life Sciences Brunel University London Uxbridge UK; ^2^ Health and Social Wellbeing, UWE Bristol UK; ^3^ Department of Health Sciences, Centre for Health and Wellbeing Across the Lifecourse Brunel University London Uxbridge UK; ^4^ Brunel Law School Brunel University London Uxbridge UK

## Abstract

This is the protocol for a Campbell systematic review. The proposed systematic review question is: What is the effectiveness of arts interventions for at‐risk and offending children and young people (8‐25 years)? There are three objectives: (1) To evaluate evidence on the effectiveness and impact of arts interventions on keeping children safe from involvement in violence and crime; (2) To synthesise evidence on factors impacting the implementation of arts interventions, and barriers and facilitators to participation and achievement of intended outcomes; (3) To develop a theory‐of‐change approach to ensure the development of an evidence‐led framework of the processes by which arts interventions might work in preventing offending behaviours.

## 1 BACKGROUND

### 1.1 The problem, condition or issue

Youth violence is recognised by the World Health Organisation (WHO) as a global public health problem that includes a range of acts from bullying and fighting through more severe sexual and physical assault to homicide (WHO, 2015). Violence amongst young people and children can lead to a range of problems including mental health issues and risk behaviours, resulting in extensive health, social and criminal justice costs. This suggests that effective prevention programmes focused on young people are needed to address a broad range of health, education and social outcomes, and that these could deliver substantial economic savings.

In the UK there is growing concern about the increase in more serious offences involving violence committed by children and young people and about growing disparities (particularly racial) in the justice system. Youth Justice Board (YJB) data for England and Wales shows that in the year April 2019 to March 2020 19,000 children aged 10 years and upwards were cautioned or sentenced in England and Wales. Children from Black and Minority Ethnic (BAME) backgrounds accounted for 32% of arrests. There has been a reduction in some forms of offending such as theft and motoring offences. However, offences relating to possession of weapons, drugs and violence have all increased, with offences involving possession of a weapon now making up 19% of all offences committed by young people who are first time entrants to the justice system (YJB/Ministry of Justice, 2021). Arts interventions have been used to divert young people from offending or other undesirable behaviours. Participatory arts programmes in community and youth justice settings can offer supportive and safe interventions that can appeal to young participants (Frater, 2019).

Arts programming in youth justice settings around the world differs in type and scope and is likely to be influenced by variations in penal policy that shape delivery, funding arrangements, experiences and outcomes from these types of programmes. These include variations in the age of criminal responsibility, which ranges from age 7 in India and certain US states through to 18 in Belgium (HAQ Centre for Child Rights, 2016). Different sentencing practices in different countries are also likely to influence arts‐based provision. For example, in England, where the age of criminal responsibility is 10 years old, children between 10 and 17 are dealt with by separate youth courts and are not sent to adult prisons. Arts provision is also likely to be influenced by framing of youth crime and the extent to which different countries focus on welfare, retributive or restorative models of justice. For example, in the Finnish system, which focuses on prevention, there is considerable overlap between the criminal justice system and the child welfare system (Marttunen, 2004). While there seems to be increasing interest in the use of arts programmes for young people in justice settings in several countries, evidence will vary in terms of scale and reporting practices.

### 1.2 The intervention

Arts interventions are diverse and this review will include interventions focused on participant involvement in artistic and creative activities such as painting, sculpting, music drama and dance. These types of arts interventions may be delivered as one‐off experiences or as a series of activities taking place over a few weeks, months or years. Arts participation may be delivered as an intervention on its own or as a ‘hook’ for other interventions, such as mentoring or education. Arts interventions may also use art as therapy (a form of psychotherapy) and as a medium to address emotional difficulties. Arts interventions will vary in terms of the settings in which they take place and will include those delivered in juvenile correctional facilities, prisons, other residential settings, dance and music studios, theatres and other community settings, schools and workplaces. Arts interventions are delivered by a range of instructors and this review will include implementation by trained professionals, volunteers, and peers.

Examples of arts interventions to be included in this review are:

- Music making
- Arts and craft, e.g., necklace making, decoupage
- Dancing
- Drama
- Film
- Podcasting
- Theatre
- Creative writing and poetry
- Photography
- Painting
- Pottery
- Sculpture
- New media/digital arts

### 1.3 How the intervention might work

Table 1 provides a preliminary logic model describing the potential chains of causes and effects of arts interventions on preventing offending and anti‐social behaviour (primary outcomes), and supporting secondary outcomes including attendance, educational attainment and psychological wellbeing. It includes consideration of intermediary outcomes associated with the costs of arts interventions for at risk and offending youth and of adverse events. Arts interventions are expected to bring about positive changes in primary and secondary outcomes through a combination of active ingredients including appropriate resources (inputs), planning and intervention activities and delivery outputs. We will consider funding models/imperatives to ensure that attention is paid to how these might impact on whether and how outcomes are successfully achieved and sustained. The logic model has been developed through discussion with the project Advisory Board and will be elaborated as the findings of the systematic review are reported and in further Advisory Board meetings.

**Table 1:** A logic model for arts interventions for at risk and offending children and young people (8–25 years)

Resources	Planning and intervention	Outputs	Outcomes	Impacts
Staffing, involvement of professionals and volunteers; financial resources; accommodation; materials. Appropriately designed activities (including therapeutic, stand alone, integrated arts) for young people considering age, gender/sex, ethnicity, socioeconomic status and educational experiences.	Operational factors in various settings. Programme planning: one‐off activities and activities of longer duration. Project management, budget, programme planning, training and supervision.	Activities, workshops, performances, exhibitions.	Primary:	Longer term:
			Offending behaviour, e.g., violence/aggression, weapon carrying/use, any other criminal activity (e.g., theft, drug offences); sexual offences, drug use/misuse; gang involvement; vandalism Anti‐social behaviours, e.g., aggression, bullying, alcohol use/misuse, problem gambling, delinquency, victimisation/harassment	Enhanced understanding of best practice, increased availability and access to arts interventions for young people at risk and in contact with criminal justice settings Wider awareness and understanding leading to policy change and systemic improvements in the justice system, education, social care and health.
			Secondary and intermediate outcomes	
			Participation/attendance at arts interventions Educational attainment, attendance and engagement (school), exclusions at school Psychological and emotional wellbeing (e.g., mood, self‐esteem, confidence, autonomy, social connections, loneliness, resilience) Costs and associated economic outcomes Adverse events (e.g., negative experiences and emotions associated with arts participation)	

### 1.4 Why it is important to do this review

Understanding what works in arts intervention programmes for preventing serious crime, violence and disruptive behaviours in at‐risk and offending children and young people (8–25 years) can support policy and intervention development. There is a need to develop an understanding of the effectiveness and impact of arts interventions on keeping children safe from involvement in violence and crime. Also central is the synthesis of evidence on factors impacting the implementation of arts interventions, and barriers and facilitators to participation and achievement of intended outcomes. This work can inform a theory‐of‐change approach using a logic model to ensure the development of an evidence‐led framework of the processes by which arts interventions might work in preventing offending behaviours. This will support the translation of evidence into accessible, useful and useable information for a range of diverse stakeholders seeking to make decisions about arts interventions, young people and offending behaviour. In this way, the work will support policy and practice to prevent young people becoming involved in violent crime.

To date, research has been characterised by a preponderance of small, short‐term studies that reveal the complexity of interventions and a variety of activities, styles and delivery formats (Anderson & Overy, 2010; Chen et al., 2016; Daykin et al., 2012). There have been few attempts to synthesise evidence across art forms, regions and countries. This review is needed because, despite the plethora of arts interventions and associated evaluation studies, there is currently no existing up‐to‐date systematic review on the effects of a full range of arts interventions for at‐risk and offending children and young people (8–25 years) on behavioural, psychosocial, cognitive and offending outcomes. This review will help develop understanding of the effectiveness of arts interventions in reducing risk and offending behaviours and build evidence on the contextual factors about how effective interventions can be best designed and implemented. It will provide an evidence‐led foundation for on‐going strategic decision making about young people, arts interventions and offending, informing policy development and practice guidelines.

## 2 OBJECTIVES

The proposed systematic review question is: What is the effectiveness of arts interventions for at‐risk and offending children and young people (8‐25 years)?

There are three objectives

- To evaluate evidence on the effectiveness and impact of arts interventions on keeping children safe from involvement in violence and crime.
- To synthesise evidence on factors impacting the implementation of arts interventions, and barriers and facilitators to participation and achievement of intended outcomes.
- To develop a theory‐of‐change approach to ensure the development of an evidence‐led framework of the processes by which arts interventions might work in preventing offending behaviours.

## 3 METHODS

### 3.1 Criteria for considering studies for this review

#### 3.1.1 Types of studies

We will include randomised and non‐randomised controlled trials and quasi‐experimental study designs. We will not include studies that did not employ a control or comparator group. We will include qualitative studies that were conducted alongside intervention trials that investigated the experiences and perceptions of participants, and that offer insight into the barriers and facilitators associated with delivering and receiving arts interventions. We will include qualitative and mixed methods studies that are focused on the delivery of an arts intervention and explored aspects of the process of intervention delivery from the perspectives of those delivering and those who are participants in the intervention and/or their carers/family members or significant agents (e.g., probation officers, victims). We will include studies from any global setting.

#### 3.1.2 Types of participants

We will include studies that include children and young people (8–25 years) who are either identified as at‐risk of offending behaviour (secondary populations) or already in the criminal justice system (tertiary populations).

#### 3.1.3 Types of interventions

We will include studies of interventions involving arts participation. Arts participation will include involvement in artistic and creative activities. Studies which include arts participation as an intervention on its own or alongside other interventions, such as mentoring, will be included. We will include studies that use art as therapy (a form of psychotherapy) and as a medium to address emotional difficulties.

Examples of arts interventions in included studies are:

- Music making
- Arts and craft, e.g., necklace making, decoupage
- Dancing
- Drama
- Film
- Podcasting
- Theatre
- Creative writing and poetry
- Photography
- Painting
- Pottery
- Sculpture
- New media/digital arts/multimedia

We will include studies that compare arts interventions to either no intervention, usual care, other types of arts intervention or non‐arts control. The intervention will involve organised arts interventions targeted to the population. We will not include associational studies between arts participation and offending behaviour.

#### 3.1.4 Types of outcome measures

##### Briefly describe the types of outcome measures that will be included and excluded

###### Primary outcomes

####### List primary outcomes

- Offending behaviour, for example, violence/aggression, weapon carrying/use, any other criminal activity (e.g., theft, drug offences); drug use/misuse; gang involvement, vandalism, sexual offences all including rates of recidivism, sexual and rearrests
- Anti‐social or pro‐social behaviours (e.g., aggression, bullying, alcohol use/misuse, problem gambling, delinquency, victimisation/harassment; sense of teamwork, belonging, worthwhileness, positive behaviours from engagement).

###### Secondary outcomes

####### List secondary outcomes

- Participation/attendance at arts interventions
- Educational attainment, attendance and engagement (school), exclusions at school
- Workplace engagement
- Psychological and emotional wellbeing (e.g., mood, self‐esteem, confidence, autonomy, social connections, loneliness, resilience)
- Costs and associated economic outcomes
- Adverse events (e.g., negative experiences and emotions associated with arts participation)

Our review will also synthesise evidence on factors impacting the implementation of arts interventions, and barriers and facilitators to participation and achievement of intended outcomes.

#### 3.1.5 Duration of follow‐up

We will consider outcomes at the following time‐points: short‐term immediately post‐intervention to <3 months; medium‐term 3 to <12 months post intervention; long‐term >1 year post intervention. Where studies report multiple follow‐ups within a single time‐point range we will preferentially extract as follows: short term, the closest follow‐up point to the end of the intervention. Medium‐ and long‐term: the latest timepoint reported.

#### 3.1.6 Types of settings

We will include studies employing arts interventions in any setting including (i) juvenile correctional facilities, prisons, other residential settings, (ii) community and workplace settings, and (iii) schools.

### 3.2 Search methods for identification of studies

#### 3.2.1 Electronic searches

We consulted Campbell guidance on searching for studies (Kugley et al., 2016). Our search strategy will include expert advice from information services experts at Brunel University London Library. We will agree an appropriate time frame for our review in discussion with the YEF and Advisory Boards. We will search relevant key databases including AMED, Academic Search Complete; APA PsycInfo; CINAHL Plus; ERIC; SocIndex; SportDiscus (via EbscoHost), Medline (via Ovid), CENTRAL, Web of Science, Scopus, PTSDPubs, Performing Arts Periodicals Database, Sage, the US National Criminal Justice Reference Service databases, the Global Policing Database, and the National Police Library.

We will search Web of Science: Conference Proceedings Citations Index and the British Library EThOS database (dissertations). We will use a combination of controlled vocabulary, i.e. medical subject headings (MeSH), and free text terms to identify published articles. In addition, we will check reference lists of reviews and retrieved articles for additional studies. An example search strategy can be found in Appendix 1 and will include separate search strings for identifying quantitative and qualitative studies. To identify the population of interest we will use the search filter proposed by the Canadian Health Libraries Association (CHLA, 2022). We will use and adapt the Cochrane highly sensitive search filter to identify randomised controlled trials (RCTs) (Higgins et al., 2021), a validated filter for identifying non‐randomised controlled studies (Waffenschmidt et al., 2020) and the University of Texas School of Public Health (University of Texas, 2022) filter for identifying qualitative studies which has been demonstrated to show good performance in sensitivity and specificity (Wagner et al., 2020).

Our searches will be worldwide and include studies from any country. We will agree the provision and support for translation of potentially relevant papers into English, and provide explicit justification for excluding non‐English papers as per MECCIR (R36).

Titles and abstracts will be independently screened by two reviewers to identify potential sources of disagreement. These will be discussed and reviewed by a third senior author in the team. Two reviewers will then screen the full texts of potentially relevant studies and apply the inclusion and exclusion criteria, with recourse to a third reviewer for any records where there is uncertainty.

We will check the references of relevant systematic reviews found in our searches.

#### 3.2.2 Searching other resources

In searching other sources we will seek expert advise. We are working with an expert Advisory Board convened by Campbell and the Youth Endowment Foundation for this purpose. We will search the WHO International Clinical Trials Registry Portal (ICTRP). We will conduct a grey literature search of databases such as Arts and Humanities Citation Index and ProQuest using our search terms. In discussion and agreement with our Advisory Board we will conduct a selected website search including the National Criminal Justice Arts Alliance and other websites with a specific focus on young people and the criminal justice system. We will conduct an Advanced Google Scholar search and sift the first 100 returns using search terms from our search strategy as appropriate. We will include reports that are authored and meet our inclusion criteria. We will use our inclusion and exclusion criteria to select grey literature. We will use our stated approach to extraction and quality assessment. We will revise our grey literature searching alongside advice from experts on the project Advisory Board.

### 3.3 Data collection and analysis

#### 3.3.1 Description of methods used in primary research

The interventions will involve organised arts interventions targeted to the population. We will not include associational studies between arts participation and offending behaviour. We will include controlled study designs. Studies that compare arts interventions to either no intervention, usual care, other types of arts intervention or non‐arts control will be included. We will also include studies comparing one type of arts intervention to another. Studies using any qualitative research method to examine context, intervention assumptions, implementation process (including barriers and facilitators), and mechanisms of impact and outcomes will be included. Qualitative studies evaluating how an arts intervention works may be conducted as independent studies or alongside controlled study designs.

#### 3.3.2 Selection of studies

Two review authors will independently assess the titles and abstracts of potential trials identified by the search strategy for their eligibility. We will obtain the full text of studies we think are eligible, or if the eligibility of a study is unclear from the title and abstract. We will exclude studies that do not match the inclusion criteria (see ‘criteria for considering studies for this review’). We will resolve disagreements between review authors regarding inclusion by discussion. If we cannot reach agreement, a third review author will assess relevant studies, and a majority decision will be made. We will not anonymise studies prior to assessment. We will include a PRISMA study flow diagram in the full review to document the screening process.

#### 3.3.3 Data extraction and management

We will frame our work with the PRISMA guidance for reporting systematic reviews (Page et al., 2021). Two review authors will independently extract data from all included studies using a standardised and piloted data extraction form. They will resolve discrepancies and disagreements by consensus. In cases where consensus cannot be achieved, a third review author will assess the article, and a majority decision will be made.

We will extract the following data from quantitative studies included in the review

- Study characteristics (aims/objectives, study design, sample size, description of the sample, country, recruitment year(s) and procedure, conflict of interest, funding source)
- Characteristics of the participants (gender/sex, age, ethnicity, socioeconomic status, education, at risk or in contact/conflict with criminal justice system)
- Description of the interventions (experimental and control), context and setting, country/location, intervention assumptions/theoretical framing, implementation processes (human and financial resources), fidelity, dose, adaptation, reach, mechanisms of impact (participant response, mediators, unanticipated consequences)
- Data collection methods including duration and timing of follow‐up/outcome assessment
- Results as outcome measures of interest to this review, including details of measurement scales and analysis methods
- Risks and biases
- Discussion including interpretations by authors, limitations and implications

We will extract the following data from qualitative studies in the review

- Study characteristics and context (aims/objectives study design, sampling approach, description of the sample, country, recruitment year(s) and procedure, conflict of interest, funding source)
- Characteristics of the participants (gender/sex, age, ethnicity, socioeconomic status, education, at risk or in contact/conflict with criminal justice system)
- Description of the interventions, context and setting, country/location, theoretical framing, implementation processes (human and financial resources), processes of impact (funding context, design and delivery model, participants' responses, unanticipated consequences)
- Data collection methods
- Findings as qualitative themes/processes including analysis methods
- Methodological limitations
- Discussion including interpretations by authors and implications

As arts interventions are complex we will extract detailed information regarding the intervention guided by the MRC guidance on process evaluations of complex interventions (Moore et al., 2015) and items on the TiDiER (Hoffmann et al., 2014) and Cert (Slade et al., 2016) checklists framed by a focus on why, what, who, where, when, how much, how well, tailoring and modifications.

#### 3.3.4 Assessment of risk of bias in included studies

One reviewer will assess the risk of bias or study quality of included studies with recourse to a 2nd reviewer where there is uncertainty. We will use the Cochrane Risk of Bias tool (Higgins et al., 2011) to evaluate included controlled trials. We will assess the following domains for each study: *Random sequence generation; Allocation concealment; Blinding of participants and providers; Blinding of assessors; Incomplete outcome data; Selective reporting; Other sources of bias*.

We will take a risk to rigour approach to evaluating qualitative studies (Noyes et al., 2018) using the CASP tool for qualitative research (CASP UK) to appraise the rigour and significance of the sampling, data, collection, analysis and reporting of results.

#### 3.3.5 Measures of intervention effect

For continuous outcome measures we will express the size of the intervention effect using the mean difference (MD) when all studies utilised the same measurement scale, or the standardised mean difference (SMD) when studies used different scales, with 95% confidence intervals. When we pool data from different scales for which the direction of interpretation varies, we will normalise the direction of the scales to a common direction. In order to aid interpretation of the pooled effect size, we will back‐transform the SMD to the most commonly used outcome scale on the basis of the median standard deviation from trials using that scale when possible.

For dichotomous outcomes we will report the Relative Risk, Odds Ratio or Risk Difference where available from individual included studies. In the event that we pool data in a de novo analysis, we will preferentially report the relative risk as the effect size of interest but will also report the risk difference.

#### 3.3.6 Unit of analysis issues

Unit‐of‐analysis issues refer to issues regarding clustering (individuals randomised/allocated in clusters), crossover designs, and studies with multiple outcome measurement time‐points.

For studies with more than two eligible active treatment groups that are included in a meta‐analysis as separate interventions, we will divide the number of participants in the control group between active treatment groups, to avoid double counting (Higgins et al., 2021). For cluster‐RCTs, we will seek direct estimates of the effect from an analysis that accounted for the cluster design. When the analysis in a cluster trial does not account for the cluster design, we will use the approximately correct analysis approach, presented in the Cochrane Handbook (Higgins et al., 2021). For cross‐over studies, we will only include data from the first phase of the study, when they are available. However, we do not anticipate finding cross‐over studies as the design is not appropriate for this research question.

#### 3.3.7 Criteria for determination of independent findings

Where we identify multiple reports for a single study we will only include data from that study once in any given analysis. Where a study reports multiple outcome domains with some conceptual overlap that fit one of our stated outcome domains, the research team will agree which of the measures conceptually best matches our outcome of interest and will only include that measure. This decision will not be made on the basis of the results of these outcomes.

#### 3.3.8 Dealing with missing data

Where there are insufficient data presented in the study report to enter into an analysis, we will request the missing data from the study authors. We will preferentially calculate and extract effect sizes derived from intention to treat analyses. We will evaluate the potential risk of bias introduced by missing data in our assessment of risk of bias, within the domain ‘Incomplete Outcome Data’ and explore the impact of risk of bias through sensitivity analyses.

#### 3.3.9 Assessment of heterogeneity

We will deal with heterogeneity by only combining studies that examine similar interventions. To estimate statistical heterogeneity, we will calculate the Chi² statistic, the between‐study variance (Tau^2^) and the proportion of this variance not due to sampling error (*I*²). We will use these measures, together with visual inspection of the forest plots to form judgements about heterogeneity. If we identify substantial heterogeneity, we will report it and explore possible causes by prespecified subgroup analysis.

#### 3.3.10 Assessment of reporting biases

We will consider the potential influence of small study biases on review findings. We will use funnel plots to visually explore small study biases where there are at least 10 included studies in a meta‐analysis.

#### 3.3.11 Data synthesis

We will conduct separate analyses of the quantitative evidence for the following comparisons: Arts intervention versus no intervention or usual practice; Arts interventions versus non‐arts control; Arts interventions versus other arts intervention.

We will pool studies of arts interventions in the primary analysis, including different types of arts, delivery mode and setting. We will use a random‐effects model to account for the anticipated heterogeneity between studies. For each comparison of interest, we will conduct separate analyses at short, medium and long‐term follow‐up. For the primary analysis, we will pool data from studies regardless of the specific population. Where there are inadequate data to enable statistical pooling we will conduct a narrative synthesis of the evidence. For head‐to‐head comparisons of different types of arts intervention, we will only pool studies if the intervention and comparators are conceptually similar.

In the event that we conduct a narrative synthesis we will separately synthesise studies within the comparisons outlined above, guided by the SWiM guideline (Campbell et al., 2020). We will first summarise all arts interventions in the primary synthesis, including different types of arts, delivery mode and setting and explore potential heterogeneity of treatment effects between studies by considering intervention setting (Custodial, community or school‐based interventions) and population age (children and adolescents 8–18 years; young adults 18–25 years.) We will report effect sizes for each reported outcome of interest with estimates of precision. We will include all relevant studies for each comparison and outcome and document the size and risk of bias of those studies in our reporting. We do not plan to present this synthesis in a tabular or graphical format.

See below for details of synthesis of qualitative research.

#### 3.3.12 Subgroup analysis and investigation of heterogeneity

Where there are adequate data and significant heterogeneity is observed in a meta‐analysis (*I* ^2^ ≥ 50%, *p* < 0.10), we will explore subgroup analyses of quantitative results by type of intervention. To explore whether there is a difference in effects between subgroups, we will use the test for subgroup differences (Deeks et al., 2020).

We will explore the following subgroups:

Intervention setting: Custodial, community or school‐based interventions.

Population Age: Children and adolescents 8–18 years; young adults 18–25 years.

We will also take an inductive approach to narratively exploring other potentially important sources of heterogeneity, for example, group vs individual therapy, the use of specific intervention characteristics such as incentives to participation, types of offending (violent, sexual, non‐violent).

#### 3.3.13 Sensitivity analysis

When sufficient data are available, we will explore the impact of risk of bias for the primary analyses, by repeating the analyses and excluding studies rated at high risk of bias.

#### 3.3.14 Treatment of qualitative research

We will take a thematic approach to analysing and synthesising data from qualitative studies. This will include line‐by‐line reading for extraction and preliminary coding, development of descriptive themes and refinement of analytical themes (see Thomas and Harden, 2008). We will conduct our thematic analysis with attention to the complexity of arts interventions for children and young people at‐risk or in the criminal justice system. We will map themes from the findings of the qualitative studies to theoretical domains of complexity relating to the intervention itself, the population, implementation of the intervention and the specific context that may impact on the process of delivering and engaging with the interventions. Table 2 outlines the complexity framework for qualitative analysis. We will take a reflexive approach and consult with Advisory Board to seek advice about relevance of themes for policy and practice.

**Table 2:** Framework for qualitative analysis

Complexity domain	Potential components
Intervention complexity	Providers, Theoretical model/assumptions, Type of art, Delivery mode/setting, Time/equipment/costs, Accessibility, youth‐focused
Contextual complexity	Residential status, Family/carer/community support, Socioeconomic factors
Population/personal complexity	Secondary or tertiary population, Values and choices, Demographics, Culture
Implementation complexity	Mode of delivery, Fidelity of intervention, Adherence, Local support structures

#### 3.3.15 Summary of findings and assessment of the certainty of the evidence

We will use the GRADE system to rank the level of certainty of the evidence (Schünemann, 2020). We will do this for both pooled effects and where we have used narrative synthesis. The GRADE approach uses five considerations (risk of bias, consistency of effect, imprecision, indirectness, and publication bias) to assess the certainty of the body of evidence for each outcome, and uses the following criteria to describe the confidence in the evidence:

- high: we are very confident that the true effect lies close to that of the estimate of the effect;
- moderate: we are moderately confident in the effect estimate; the true effect is likely to be close to the estimate of effect, but there is a possibility that it is substantially different;
- low: our confidence in the effect estimate is limited; the true effect may be substantially different from the estimate of the effect;
- very low: we have very little confidence in the effect estimate; the true effect is likely to be substantially different from the estimate of effect.

We will decrease the grade rating by one (−1), two (−2), or three (−3) levels, up to a maximum of −3, (or very low) for any criteria, based on the level of concern it raises.

We will use GRADE CERQUAL to asses our confidence in the qualitative evidence. CERQual has four components (methodological limitations, relevance, adequacy and coherence) and uses the following criteria for judging confidence in the body of literature (Lewin et al., 2015)

methodological limitations—the extent to which there are problems in design or conduct of primary studies that contributed evidence to the review

relevance—the extent to which evidence in primary study is applicable (perspective, population, phenomenon of interest, setting)

coherence—the degree to which primary studies provide convincing explanations for patterns

adequacy—the degree of richness and quantity/scope of data

Confidence will be decreased if there are serious or very serious limitations in the design or conduct of the study, the evidence is not relevant to the study objectives, the findings/conclusions are not supported by the evidence or the data are of inferior quality and inadequate in supporting the findings. Confidence will be increased if the study is well designed with few limitations, the evidence is applicable to the context specified in the objectives, the findings/conclusions are supported by evidence and provide a convincing explanation for any patterns found or the data supporting findings are rich and of high quality.

## CONTRIBUTIONS OF AUTHORS

Please give brief description of content and methodological expertise within the review team. The recommended optimal review team composition includes at least one person on the review team who has content expertise, at least one person who has methodological expertise and at least one person who has statistical expertise. It is also recommended to have one person with information retrieval expertise.

Who is responsible for the below areas? Please list their names:

- Content: Louise Mansfield, Norma Daykin and Louise Forde
- Systematic review methods: Neil O'Connell
- Statistical analysis: Daniel Bailey
- Information retrieval: Robyn Smith, Jake Gifford, and Garcia Ashdown‐Franks.

## DECLARATIONS OF INTEREST

Please declare any potential conflicts of interest. For example, have any of the authors been involved in the development of relevant interventions, primary research, or prior published reviews on the topic?

None.

## PRELIMINARY TIMEFRAME

Approximate date for submission of the systematic review.

April 2023.

## PLANS FOR UPDATING THIS REVIEW

Review will be updated if there is an identified need and funding available.

## SOURCES OF SUPPORT

**Internal sources**

- Brunel University London Information Services, UK

Support for database searching

**External sources**

- Youth Endowment Foundation, UK

Funding support and secretariat for Advisory Board

## Supporting information

Supporting information.
