# Supplementary material for: PROTOCOL: A mixed methods systematic review on the effects of arts interventions for at‐risk and offending children and young people on behavioural, psychosocial, cognitive and offending outcomes
Source: Campbell Syst Rev. 2023 Jan 10;19(1):e1298. doi: 10.1002/cl2.1298 (PMC9831279; doi:10.1002/cl2.1298)
Supplement: Supplementary file 1 — Supporting information. [file CL2-19-e1298-s001.docx]

## 1 Ovid MEDLINE(R) ALL search strategy

**Ovid MEDLINE**

**Quantitative studies search strategy**

1. EXP/adolescent
2. (teen* OR youth* OR adolescen* OR juvenile* OR (young ADJ2 (adult* OR person* OR individual* OR people* OR population* OR man OR men OR wom#n)) OR youngster* OR first-grader* OR second-grader* OR third-grader* OR fourth-grader* OR fifth-grader* OR sixth-grader* OR seventh-grader* OR highschool* OR college* OR ((secondary OR high*) ADJ2 (school* OR education ))).
3. 1 or 2
4. (*offend* OR delinq* OR crim* OR prison* OR * inmate* OR exclu* adj3 school OR exclu* adj3 educ* OR disadvantaged).ab,ti
5. 3 AND 4
6. (Music* OR sing* OR rap* OR grime OR drill music OR hip*hop OR drumm* OR choral OR choir OR Art* OR Danc* OR drama* OR creative OR creative writ* OR poet* OR spoken word OR perform* OR Photog* OR Paint* OR drawing OR Pottery OR ceramic OR sculpt OR craft* OR perform* OR film OR video OR digit* OR theatr* OR podcast OR biogr* OR graffiti OR mural OR digital storytelling OR visual media OR colouring OR crochet* OR knitting OR *zine OR fashion).ab,ti
7. Randomised controlled trial.pt
8. Controlled clinical trial.pt
9. Randomized.ab
10. Placebo.ab
11. trial.ti
12. 7 OR 8 OR 9 OR 10 OR 11
13. EXP animals/ NOT humans.sh
14. 12 NOT 13
15. EXP/ cohort studies/OR exp epidemiologic studies/OR exp clinical trial/OR exp evaluation studies as topic/OR exp statistics as topic/
16. ((control and [group* OR study]) OR (time AND factors) OR program OR survey* OR ci OR cohort OR comparative stud*OR evaluation studies OR follow-up*).mp.
17. 15 OR 16
18. (animals/not humans/) or comment/or editorial/or expreview/or meta analysis/or consensus/or exp guideline/
19. hi.fs. or case report.mp.
20. 18 OR 19
21. 17 NOT 20
22. 14 OR 21
23. 5 AND 6 AND 22

**Qualitative studies search strategy**

EXP/adolescent

2. (teen* OR youth* OR adolescen* OR juvenile* OR (young ADJ2 (adult* OR person* OR individual* OR people* OR population* OR man OR men OR wom#n)) OR youngster* OR first-grader* OR second-grader* OR third-grader* OR fourth-grader* OR fifth-grader* OR sixth-grader* OR seventh-grader* OR highschool* OR college* OR ((secondary OR high*) ADJ2 (school* OR education))).

3. 1 or 2

4. (*offend* OR delinq* OR crim* OR prison* OR * inmate* OR exclu* adj3 school OR exclu* adj3 educ* OR disadvantaged).ab,ti

5. 3 AND 4

6. (Music* OR sing* OR rap* OR grime OR drill music OR hip*hop OR drumm* OR choral OR choir OR Art* OR Danc* OR drama* OR creative OR creative writ* OR poet* OR spoken word OR perform* OR Photog* OR Paint* OR drawing OR Pottery OR ceramic OR sculpt OR craft* OR perform* OR film OR video OR digit* OR theatr* OR podcast OR biogr* OR graffiti OR mural OR digital storytelling OR visual media OR colouring OR crochet* OR knitting OR *zine OR fashion).ab,ti

7. exp/ Qualitative research

8. (((semi-structured or semistructured or unstructured or informal or in-depth or indepth or face-to-face or structured or guide) adj3 (interview* or discussion* or questionnaire*))).ab.ti

9. (focus group* or qualitative or ethnograph* or fieldwork or field work or key informant).ab.ti

10. interviews as topic/ or focus groups/ or narration/ or qualitative research/

11. 7 OR 8 OR 9 OR 10

12. 5 AND 6 AND 11
